# Rezafungin as Primary Prophylaxis of *Pneumocystis jirovecii* Pneumonia in a Critically Ill Person Presenting with AIDS with Trimethoprim/Sulfamethoxazole Allergy: A Case Report

**DOI:** 10.3390/jof12030189

**Published:** 2026-03-05

**Authors:** Martina Bottanelli, Alice Mulè, Chiara Molteni, Martina Gerbi, Mauro Pietro Zago, Sara Volpi, Sofia Pettenuzzo, Alessandro Pandolfo, Valentina Morena, Nicole Gemignani, Michele Fogliata, Federico Conti, Alessandra Consonni, Lucia Bradanini, Silvia Pontiggia, Stefania Piconi

**Affiliations:** 1 Infectious Diseases Department, Alessandro Manzoni Hospital—ASST Lecco, 23900 Lecco, Italy; m.bottanelli@asst-lecco.it (M.B.); a.mule@asst-lecco.it (A.M.); c.molteni@asst-lecco.it (C.M.); m.gerbi@asst-lecco.it (M.G.); s.volpi@asst-lecco.it (S.V.); s.pettenuzzo@asst-lecco.it (S.P.); a.pandolfo@asst-lecco.it (A.P.); v.morena@asst-lecco.it (V.M.); ni.gemignani@asst-lecco.it (N.G.); fe.conti@asst-lecco.it (F.C.); s.pontiggia@asst-lecco.it (S.P.); 2 Emergency Surgery Department, Alessandro Manzoni Hospital—ASST Lecco, 23900 Lecco, Italy; m.zago@asst-lecco.it (M.P.Z.); m.fogliata@asst-lecco.it (M.F.); 3 Clinical Microbiology Department, Alessandro Manzoni Hospital—ASST Lecco, 23900 Lecco, Italy

**Keywords:** rezafungin, *Pneumocystis jirovecii*, primary prophylaxis, AIDS presenter, PWH, long-acting, TMP/SMX hypersensitivity, off-label, case report, echinocandins

## Abstract

Primary prophylaxis of *Pneumocystis jirovecii* pneumonia (PCP) in people with HIV (PWH) and CD4+ counts <200 cells/µL using trimethoprim/sulfamethoxazole (TMP-SMX) is highly effective but often poorly tolerated. Alternative agents may have limited efficacy or availability. Although rezafungin demonstrated PCP protection comparable to TMP-SMX in animal models, human data are limited to the ongoing ReSPECT trial, and evidence in PWH is lacking. We report the first use of rezafungin as PCP prophylaxis in a PWH. A 43-year-old man presenting with AIDS (HIV-RNA 8.48 × 10^6^ copies/mL; CD4+ 20 cells/µL) was admitted with disseminated tuberculosis and multiple bowel perforations requiring urgent surgery. The postoperative course was marked by infectious and surgical complications. Antitubercular therapy and TMP-SMX prophylaxis were initiated postoperatively, followed by antiretroviral therapy (ART). Later, TMP-SMX was discontinued due to hypersensitivity. Because drug–drug interactions precluded atovaquone or dapsone and pentamidine was unavailable, rezafungin was started. No adverse events or fungal breakthrough infections occurred despite abdominal complications. Further data are needed to determine whether rezafungin represents a viable prophylactic option when standard agents are contraindicated or unavailable.

## 1. Introduction

*Pneumocystis jirovecii* pneumonia (PCP) is one of the most frequent and life-threatening opportunistic infections among people with HIV (PWH) with CD4+ count <200 cell/ µL [1]. Before the availability of ART and PCP prophylaxis, PCP incidence reached 70–80% among people with AIDS and was associated with high mortality (20–40%). The risk was highest in individuals with CD4+ <200 cells/µL or less than 14% of total lymphocytes, reaching up to 20 cases per 100 person-years. With the widespread use of ART and systematic prophylaxis, PCP incidence has declined markedly: contemporary cohort studies report first-episode PCP rates of fewer than 10 cases per 1000 person-years among virologically suppressed PWH and CD4+ 100–200 cells/µL, but the risk remains higher in those with lower CD4+ counts or uncontrolled viremia [2,3].

First-line prophylaxis is well established with trimethoprim/sulfamethoxazole (TMP-SMX) due to its efficacy, accessibility, cross-protection against other pathogens such as *Toxoplasma gondii*, and concomitant broad-spectrum antibacterial activity [1,2,3,4,5,6,7]. However, the use of TMP-SMX may be limited by hypersensitivity, hematological and renal toxicity, and drug–drug interactions. Specifically, hypersensitivity reactions such as maculopapular rash to TMP/SMX are relatively common among PWH, occurring in up to 40% of cases, although severe reactions are less common [4,8,9]. Alternative PCP prophylactic agents such as dapsone, atovaquone, and aerosolized pentamidine are indicated as second-line options, but they have some limitations, such as reduced prophylactic effectiveness compared with TMP/SMX, limited availability in hospital pharmacies, no efficacy against toxoplasmosis, and some pharmacologic interactions (i.e., with rifampicin or rifabutin) [2].

Rezafungin, a next-generation echinocandin with a prolonged half-life (once weekly intravenous administration) and favorable safety profile [10,11], has shown promising results in murine models for PCP prophylaxis, demonstrating similar efficacy to TMP-SMX with fewer adverse effects and drug interactions [10,11,12,13,14]. The only currently available clinical data derive from the ongoing “Study of Rezafungin Compared to Standard Antimicrobial Regimen for Prevention of Invasive Fungal Diseases in Adults Undergoing Allogeneic Blood and Marrow Transplantation (ReSPECT trial, NCT04368559)”, a phase 3, multicentric, randomized, double-blind study comparing rezafungin to standard antimicrobial regimens (TMP-SMX and azoles like fluconazole and posaconazole) for the prevention of invasive fungal diseases (PCP, invasive candidiasis and aspergillosis) in patients undergoing allogeneic hematopoietic stem cell transplantation. To date, no published clinical data exist on the use of rezafungin for PCP prophylaxis in PWH [2].

We report the first known case of off-label use of rezafungin as primary prophylaxis for PCP in a critically ill person with AIDS with no available standard prophylactic options.

## 2. Case Report

On hospital day (HD) 1 at the end of 2024, a 43-year-old Italian man was admitted at the Infectious Diseases Department of Alessandro Manzoni Hospital, Lecco, Italy, after presenting to the Emergency Room with profuse diarrhea, high-grade fever, marked weight loss, and generalized lymphadenopathy. The HIV test performed at admission resulted positive, measured using the HIV Ag/Ab Combo Reagent Kit (Abbot) chemiluminescent microparticle immunoassay (CMIA) on the Alinity i automated platform (Abbott, Abbott Laboratories, Abbott Park, IL, USA). At diagnosis, HIV-1 RNA was 8,480,000 copies/mL, and CD4+ count was 20 cells/µL (4%); serum cryptococcal antigen test was negative and Quantiferon TB test was positive (TB2 tube only, value: 2.72 IU/mL). Additionally, the patient was seronegative for syphilis, hepatitis B (HbsAg, HBcAb and HBsAb), and hepatitis C, while he had a past exposure (IgG+/IgM−) to HAV, CMV, and *T. gondii*. Serological testing was performed using the following assays: Syphilis TP Reagent Kit (Abbott) chemiluminescent immunoassay CLIA; anti-HCV Reagent Kit (Abbott) CMIA; HBV Reflex panel (Anti-HBs, HBsAg Qualitative II, Anti-HBc II, Anti-HBc IgM, Anti-HBe, and HBeAg; CMIA on Alinity i, Abbott); LIAISON CMV IgM II and IgG II and LIAISON Toxo IgM II and IgG II (Diasorin, Saluggia, Italy), with both CMIA on the Liaison XL platform.

A total-body contrast-enhanced CT scan revealed extensive lymphadenopathy (laterocervical, mediastinal, mesenteric), with features of central necrosis and colliquation. Pulmonary imaging showed apical confluent pseudonodular consolidations, a cavitating lesion in the right upper lobe, and a diffuse “tree-in-bud” pattern. In the abdomen, segmental ileal and jejunal wall thickening with radiological signs of perforation were described.

On HD 4, the patient underwent an urgent exploratory laparotomy, where three small bowel perforations were found that led to a wide resection of the small intestine. Histology confirmed transmural necrosis and granulomatous inflammation. In the immediate postoperative period, the patient necessitated invasive mechanical ventilation and vasopressor support. From peritoneal fluid cultures grew *Enterobacter cloacae*, methicillin-sensitive *Staphylococcus aureus*, and fluconazole-sensitive *Candida albicans*. Bacterial and yeast isolates were identified using matrix-assisted laser desorption/ionization time-of-flight mass spectrometry (MALDI-TOF MS) with the VITEK MS system (bioMérieux, Marcy-l’Étoile, France). Antimicrobial susceptibility testing (AST) was performed using the automated VITEK 2 system (bioMérieux, Marcy-l’Étoile, France). Based on these findings, ongoing antimicrobial therapy started on HD 4 with piperacillin/tazobactam, vancomycin, and fluconazole was continued for 10 days. Two days after the first surgery, a second look was performed, resulting in resection of an additional small bowel segment and two anastomoses (ileo-colic and colo-colic) with a protective ileostomy were constructed, along with fascial closure.

Upon subsequent onset of respiratory failure associated with CT evidence of pseudonodular consolidations exhibiting a perbronchial “tree-in-bud” distribution, diagnostic bronchoscopy was performed. Analysis of bronchoalveolar lavage (BAL) specimens revealed negative standard cultures, galactomannan testing, and polymerase chain reaction (PCR) for *P. jirovecii.* Detection of *P. jirovecii* DNA was performed using the ELITe InGenius SP200 (ELITechGroup, Puteaux, France), a fully automated sample-to-result real-time PCR system integrating nucleic acid extraction, amplification, and detection. Concurrently, microscopy for acid-fast bacilli and PCR for *Mycobacterium tuberculosis complex* were positive on BAL, pleural fluid, and a sampled abdominal lymph node. Thereafter, mycobacterial culture confirmed a fully pansensitive strain.

On HD 5, standard quadruple intravenous antitubercular therapy (rifampin, isoniazid, pyrazinamide, ethambutol) was started. Moreover, CMV DNA positivity on BAL led to the initiation of antiviral therapy with ganciclovir, continued for 21 days. Quantitative detection of CMV DNA was performed using the ELITe InGenius SP200 (ELITechGroup, Puteaux, France), a fully automated sample-to-result real-time PCR platform. At the same time, PCP and *T. gondii* prophylaxis were started with TMP-SMX thrice a week. Prophylaxis was initiated considering prior exposure to *T. gondii* and irrespective of negative *P. jirovecii* colonization status, given the low CD4+ count, in accordance with the current guidelines [4]. Given the patient’s critical status, after a lumbar puncture excluded central nervous system (CNS) involvement by *M. tuberculosis* and cryptococcal meningitis, ART was introduced early on HD 11 using emtricitabine (200 mg)/tenofovir alafenamide (25 mg) once daily plus dolutegravir 50 mg twice daily, adjusted for co-administration with rifampin.

After an initial recovery, the clinical course was complicated by severe septic shock and respiratory failure, necessitating Intensive Care Unit re-admission with invasive mechanical ventilation, vasopressor support, and broad-spectrum antimicrobial therapy with meropenem, vancomycin, and caspofungin, which continued for 10 days, leading to a rapid clinical recovery.

On surgical indication, from HD 60 to HD 75, a chyme reinfusion system was implemented through the ileostomy to optimize nutritional absorption and reduce parenteral nutrition dependence. On the following days, a significant increase in ileostomy output was observed, raising concerns for intestinal malabsorption and possible bacterial translocation. Empirical antibiotic therapy with tigecycline [given the rectal colonization by vancomycin-resistant *Enterococcus faecium* (VRE)] and ceftazidime was initiated on HD 67 and continued for 11 days. Simultaneously, as a marker for potential fungal translocation or early invasive fungal infection, serum β-D-glucan levels were monitored (17.7 pg/mL on HD 67 and 9.18 pg/mL after 2 weeks), using Toxinometer MT 6500 (Alifax, Polverara, Italy) with negative plasma PCR for *Candida* spp. (Cand-ID Real Time PCR). Since the patient remained clinically stable and afebrile, no antifungal therapy was introduced.

On approximately HD 80, the patient developed a widespread severe pruritic erythematous rash with eosinophilia, strongly suggestive of a hypersensitivity reaction to TMP-SMX, leading to its discontinuation after 75 days of use. Subsequently, the symptomatology disappeared, and eosinophil count normalized. The immuno-virological profile on HD 86 was as follows: HIV-1 RNA undetectable; CD4+ count 70 cells/µL (10%). Due to the significant drug–drug interactions between rifampin-based antitubercular therapy and second-line options for PCP prophylaxis (atovaquone, dapsone) and the unavailability of inhaled pentamidine, we decided to start an off-label antifungal prophylaxis with rezafungin (loading dose 400 mg on HD 93, followed by 200 mg IV weekly). The regimen was well-tolerated, without infusion-related reactions, hepatic toxicity, or evidence of breakthrough fungal infections, providing an effective alternative PCP prophylaxis strategy during immunosuppression.

After clinical stabilization and nutritional state improvement, surgical closure of the ileostomy was performed on HD 128. However, a new intestinal perforation occurred after 2 days, resulting in further resection and subsequent intestinal recanalization being required. Cultures from peritoneal fluid and urine during this septic episode yielded VRE. Treatment with tigecycline and ceftazidime was administered for 10 days. Throughout this period, rezafungin was maintained as prophylaxis for invasive fungal infections, providing broad-spectrum antifungal coverage and eliminating the need for adjunctive antifungal molecules.

Notably, no evidence of an invasive fungal infection emerged. This was supported by the absence of clinical signs suggestive of fungal disease, repeatedly negative blood cultures, low serum β-D-glucan levels, and negative plasma PCR testing for *Candida* spp.

**Figure 1:**
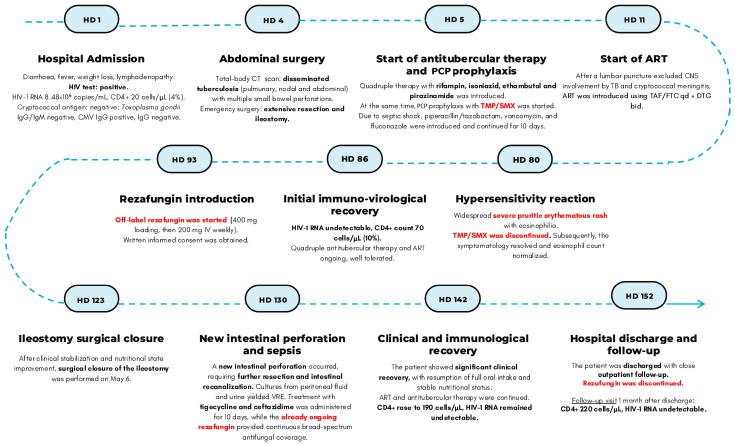
Timeline of the main clinical events and therapeutic changes. Abbreviations: HD, hospital day; IgG, immunoglobulin *G*; IgM, immunoglobulin *M*; CMV, cytomegalovirus; CT, computed tomography; *PCP*, *Pneumocystis jirovecii* pneumonia; CNS, central nervous system; *TB*, tuberculosis; *ART*, antiretroviral therapy; TAF/FTC, tenofovir alafenamide/emtricitabine; qd, once daily; DTG, dolutegravir; bid, twice daily; TMP/SMX, trimethoprim/sulfamethoxazole; VRE, vancomycin-resistant Enterococcus faecium.

By late May, the patient showed significant clinical recovery, with resumption of full oral intake and stable nutritional status. ART and antitubercular therapy were continued. CD4+ count rose to 190 cells/µL, and HIV-1 RNA remained undetectable. Rezafungin prophylaxis was maintained weekly until discharge on HD 152. The timeline of the main clinical events and therapeutic changes is reported in Figure 1.

## 3. Discussion

To our knowledge, this is the first documented case of rezafungin being used as PCP prophylaxis in PWH. The decision to start rezafungin was based on the complete unavailability of standard prophylactic agents. Specifically, given the documented allergy to TMP-SMX, the pharmacological incompatibility between atovaquone or dapsone and concomitant rifampicin-based antitubercular treatment, and the lack of access to aerosolized pentamidine, rezafungin was used as a salvage strategy in a critically ill person with AIDS (Table 1).

Echinocandins primarily target the β-(1,3)-D-glucan-rich cell wall of *Pj* asci and have limited or no direct activity against the trophic forms, which represent the metabolically active stage responsible for alveolar invasion. Despite this, echinocandins can play a role in PCP prophylaxis, where inhibition of ascus formation and transmission may be sufficient to prevent progression to clinical disease [15].

Previous clinical experience with echinocandins in PCP has been reported with agents such as caspofungin and anidulafungin, mainly as salvage or adjunctive therapy for established PCP. Available evidence consists largely of case reports and case series, often describing echinocandins used in combination with TMP/SMX or pentamidine, with heterogeneous outcomes [2,16].

Preclinical studies conducted in murine models have demonstrated the potential of echinocandins, including rezafungin, for the prevention of PCP. These studies suggest that rezafungin may offer comparable efficacy to the standard prophylactic agent (TMP-SMX), with the added benefit of improved tolerability [12,13,14].

Despite the promising preclinical results, clinical evidence in humans remains limited, with the only available data coming from the ongoing ReSPECT trial, which is still in the recruitment phase. This phase 3, multicentric, randomized, double-blind study aims to compare rezafungin to standard antimicrobial regimens (TMP-SMX and azoles like fluconazole and posaconazole) for the prevention of invasive fungal diseases (PCP, invasive candidiasis and aspergillosis) in patients undergoing allogeneic blood and marrow transplantation. To date, no clinical data are available regarding the use of rezafungin in PWH, leaving a gap in understanding its potential in this high-risk population [2].

In our case, rezafungin was well-tolerated, with no adverse events or clinically significant drug–drug interactions, despite the patient’s frailty and polypharmacy. This suggests a potentially favorable safety profile in PWH with a complex clinical condition, particularly when standard prophylactic agents are contraindicated. Notably, the patient experienced no signs of PCP or other fungal infections during the follow-up. After PCR for *P. jirovecii* was negative at first BAL, no further diagnostic testing was performed during hospitalization, as the patient never showed clinical or radiological findings suggestive of PCP. Rezafungin was used exclusively as primary prophylaxis considering the low CD4+ count, according to current guidelines.

Given the observational nature of this single case and the limited duration of rezafungin exposure, the absence of PCP occurrence during follow-up cannot be directly interpreted as a protective effect of the prophylaxis. Rather, we have described the feasibility and tolerability of rezafungin as an off-label, salvage prophylactic strategy in a critically ill PWH with advanced infection, in whom all standard prophylactic options were either contraindicated or unavailable.

The choice of rezafungin was driven by its pharmacokinetic and pharmacodynamic properties, including prolonged systemic and tissue exposure, high lung penetration, and preclinical evidence specifically supporting its activity against *P. jirovecii* in murine models. In addition, rezafungin was preferred in the context of marked polypharmacy in a critically ill and surgically complex patient, with the aim of minimizing the overall infusion burden and reducing treatment complexity.

In our case, the prolonged hospitalization was mainly related to surgical complications and repeated septic episodes rather than delayed immunologic recovery. Although immune reconstitution was closely monitored, it did not represent the primary driver of hospitalization duration. As the patient had achieved satisfactory immunovirological recovery by the time of discharge, outpatient continuation was not required, but rezafungin would have represented a feasible option for ongoing PCP in the absence of oral alternatives.

A known limitation of using echinocandins such as rezafungin in PWH is their lack of prophylactic activity against *T. gondii* [2,10], a drawback shared by other second-line PCP prophylactic agents like dapsone (which requires combination with pyrimethamine for *T. gondii* prophylaxis) and inhaled pentamidine [1,2,3,4]. In our case, in the absence of effective and safe options for *T. gondii* prophylaxis, considering the increase in the CD4+ count, the undetectable viral load, and the strict adherence to ART, we opted for close clinical and laboratory monitoring in an inpatient setting.

An interesting observation was the occurrence of a second gastrointestinal perforation during the ongoing rezafungin PCP prophylaxis. This event took place during immunosuppression and significant mucosal vulnerability, conditions that typically predispose to bacterial and fungal invasive infections. Thanks to its well-known broad-spectrum antifungal activity and its efficacy in prevent fungal invasive candidiasis [17,18], rezafungin allowed for effective control of the patient’s abdominal condition (no clinical or microbiological evidence of fungal superinfections) and spared introducing any additional antifungal agent.

## 4. Conclusions

In conclusion, this case highlights the potential role of rezafungin as a salvage prophylactic agent for PCP in PWH when standard therapies are contraindicated or unavailable. However, the lack of *T. gondii* coverage remains a limitation.

In our case, rezafungin was safe and compatible with multidrug therapy. Although its efficacy cannot be established from a single clinical outcome, no PCP or other invasive fungal infections occurred during rezafungin treatment, despite severe abdominal complications and the markedly low CD4+ count in this patient with AIDS.

Supported by preclinical data and the ReSPECT trial, rezafungin may represent a safe prophylactic option for PCP when standard agents are contraindicated or unavailable. While this off-label use appears promising, clinical evidence remains limited, and further studies are needed to define its place in the prophylactic management of PCP in PWH.

## Figures and Tables

**Table 1:** Comparison of available PCP prophylactic options in PWH and rationale for rezafungin use. Abbreviations: TMP-SMX, trimethoprim/sulfamethoxazole; PCP, *Pneumocystis jirovecii* pneumonia; IV, intravenous; CYP, Cytochrome P450.

Agents	Route	Anti-PCP Efficacy	Activity vs. *T. gondii*	Drug–Drug Interaction with Rifampin	Mechanism of Interaction	Main Limitations	Applicability in This Case
TMP-SMX	Oral	High (first-line) [4,5]	Yes [4,5]	No [4,5,6]	_	Hypersensitivity, renal and hematological toxicity, hyperkalemia [4,5,7]	Severe allergic reaction
Dapsone	Oral	Moderate [4,5]	No (needs pyrimethamine) [4,5]	Yes [4,5,6]	CYP3A4 and CYP2C induction: dapsone plasma levels reduction [4,5,6]	Hemolysis, methemoglobinemia [4,5]	Rifampin interaction
Atovaquone	Oral	Moderate [4,5]	Yes (alternative option, often combined with pyrimethamine) [4,5]	Yes (major) [4,5,6]	Strong induction of hepatic metabolism and reduced enterohepatic circulation [4,5,6]	Malabsorption, availability [4,5]	Malabsorption + rifampin interaction
Pentamidine (aerosol)	Inhaled	Moderate [4,5]	No [4,5]	No [4,5,6]	_	Limited availability	Not available
Pentamidine (IV)	IV (q3–4 weeks)	Moderate [4,5]	No [4,5]	No [4,5,6]	Unknown (antiprotozoal)	Nephrotoxicity, hypoglycemia/hyperglycemia, QT prolongation, limited availability	Not available
Rezafungin	IV (weekly)	Preclinical efficacy [12,14]	No	No clinically relevant interaction	Non-CYP metabolism, no enzyme induction [10,12]	Off-label, IV only	Only viable option

## Data Availability

The original contributions presented in this study are included in the article. Further inquiries can be directed to the corresponding author.

## Abbreviations

AIDS	Acquired immunodeficiency syndrome
ART	Antiretroviral therapy
BAL	Bronchoalveolar lavage
Bid	Twice daily
CMV	Cytomegalovirus
CNS	Central nervous system
CT	Computed tomography
CYP	Cytochrome P450
DTG	Dolutegravir
FTC	Emtricitabine
HAV	Hepatitis A virus
HBcAb	Hepatitis B core antibody
HBsAb	Hepatitis B surface antibody
HBsAg	Hepatitis B surface antigen
HCV	Hepatitis C virus
HIV	Human immunodeficiency virus
HIV-1 RNA	Human immunodeficiency virus type 1 ribonucleic acid
IgG	Immunoglobulin G
IgM	Immunoglobulin M
IV	Intravenous
PCR	Polymerase chain reaction
PCP	*Pneumocystis jirovecii* pneumonia
PWH	People with HIV
qd	Once daily
TB	Tuberculosis
TAF	Tenofovir alafenamide
TMP-SMX	Trimethoprim/sulfamethoxazole
VRE	Vancomycin-resistant Enterococcus faecium
